# The Impact of Gluten Friendly Flour on the Functionality of an Active Drink: Viability of *Lactobacillus acidophilus* in a Fermented Milk

**DOI:** 10.3389/fmicb.2018.02042

**Published:** 2018-08-30

**Authors:** Barbara Speranza, Antonio Bevilacqua, Daniela Campaniello, Milena Sinigaglia, Daniela Musaico, Maria R. Corbo, Carmela Lamacchia

**Affiliations:** Department of the Science of Agriculture, Food and Environment, University of Foggia, Foggia, Italy

**Keywords:** synbiotic, Gluten Friendly, acidification, shoulder length, desirability

## Abstract

The Gluten Friendly^TM^ Technology is an innovative method that induces structural changes in gluten proteins. In this paper a synbiotic fermented milk, containing *Lactobacillus acidophilus* La-5 and Gluten Friendly Flour (GFF), was proposed. A mixture design was used to combine flour, temperature and probiotic to study the effects of these variables on the acidification. The experiments were done on both GFF and control flour (CF). Thus, the following conditions were chosen to produce the fermented milk: *L. acidophilus* at 6.5 log cfu/ml; flour at 2.5 g/l; temperature at 37°C. Then, the fermented milk was produced and stored at 4°C for 90 days. The most important result was the positive effect of GFF on the viability of the probiotic, with a prolongation of the shoulder length to 20 days (12–13 days in the control). Moreover, GFF did not act on the sensory scores and on the physico-chemical parameters.

## Introduction

Today the demand for healthy products is continuously increasing and the food industry has been showing interest and marketing functional foods, i.e., foods able to provide health benefits as they include basic nutrients and compounds reducing the risk of several diseases (González Fabre, 2008). Synbiotic foods are generally considered functional foods because they beneficially affect the host by improving the survival and implantation of probiotic microorganisms in the gastrointestinal tract by selectively stimulating their growth and/or the metabolism (Cencic and Chingwaru, 2010). Even if there is not a general consensus toward the definition of synbiotic foods, they could be defined as products containing a combination of probiotics and prebiotics that can act synergistically to modulate the intestinal microbiota and positively impact on people’s health (Gotteland, 2010). So that probiotics exert health effects, the recommended minimum level of viable cells has been suggested to be between 10^6^–10^7^ cfu/ml at the moment of consumption (Silva et al., 2015) and prebiotics have shown good results in helping probiotics to maintain their viability and functionality throughout all food processing steps (Akin et al., 2007; Cruz et al., 2009).

FAO/WHO (2006) defines “*prebiotics as a non-viable food component that confer health benefit(s) on the host associated with modulation of the microbiota*”; in general, prebiotics are carbohydrate ingredients of different origin: breast milk, soybeans, inulin sources (like Jerusalem artichoke, chicory roots etc.), raw oats, unrefined wheat, unrefined barley, yacon, and non-digestible oligosaccharides [oligofructose, and (trans) galacto-oligosaccharides (GOS)(Pokusaeva et al., 2011)]. Recently other sources (ß-glucans, ulvan, etc.) have been explored for their potential benefits as prebiotics (Saulnier et al., 2009) and this remains an active area of research. From a technological point of view, the addition of prebiotic to foods has been demonstrated to improve sensory characteristics such as taste and texture (Al-Sheraji et al., 2013); some evidences of enhancements of the stability of foams, emulsions, and mouthfeel in a vast range of food applications like dairy and baking products have been also reported (Al-Sheraji et al., 2013).

In 2013 a new and innovative method (Gluten Friendly^TM^) has been developed (PCT/IB2013/000797) (Lamacchia et al., 2013, 2015); it consists into the application of microwave energy for few seconds to hydrated wheat kernels. Due to some structural modifications to endosperm components, the immunogenicity of the most common epitopes involved in celiac disease was reduced *in vitro* (Lamacchia et al., 2016), but the nutritional and technological properties necessary to process flour into bread, pasta, and other baked goods remain unchanged. Additional researches (Bevilacqua et al., 2016b) found that bread produced with Gluten Friendly Flour (GFF) was able to modify the qualitative-quantitative composition of gut microbiota (bifidogenic effect and on the growth of lactobacilli in the gut microbiota in a complex system).

The probiotic potential of *L. acidophilus* La-5 was investigated and assessed in the past. Brasili et al. (2013) found that the supplementation of this probiotic induced a positive modulation of urinary and fecal metabolic profiling thus suggesting a possible effect on the prevention and/or the reduction of age-related metabolic dysfunction, while Zarrati et al. (2013) reported a significant modulation on T-cell subset specific gene expression in peripheral blood mononuclear cells among overweight and obese individuals. Other probiotic effects include the inhibition of *Escherichia coli* O157:H7 (Medellin-Peñna and Griffiths, 2009), and the reduction of *Streptococcus mutans* in saliva (Bafna et al., 2018).

The strain can be successfully inoculated and supplemented with different foods, among others fruit-based ice-cream (Senanayake et al., 2013), blue cheese (Zadernowska et al., 2015), or loaded in microcapsules (Gebara et al., 2013; Ranadheera et al., 2015).

A preliminary research (Bevilacqua et al., 2016b) showed the potential of the combination of GF products with *L. acidophilus*, as the survival time of the probiotics was prolonged by 15–20 h at 37°C and in a minimal medium.

Considering all these data, the aim of the present study was to design a synbiotic milk, fermented with *L. acidophilus* La-5 and containing GFF as a beneficial component to improve the viability of the probiotic in real conditions and design a new functional food with an improved survival of *L. acidophilus* La-5. The research was divided into two different steps: (1) a product optimization to choose the optimal conditions to design the active drink, in terms of level of inoculum, temperature and amount of GFF; (2) validation at laboratory level with the production of a functional fermented milk and the evaluation of its microbiological, physico-chemical and sensorial quality during refrigerated storage for 90 days.

## Materials and Methods

### Product Optimization

#### Microorganism, Flour and Milk

*Lactobacillus acidophilus* La-5 was purchased from Chr. Hansen (Hørsholm, Denmark). Prior each assay, the strain was grown in MRS broth (Oxoid, Basingstoke, United Kingdom) at 37°C for 24 h and then centrifuged at 4000 *g* for 10 min; the supernatant was discarded, and the pellet was suspended in sterile distilled water.

The GFF was prepared as follow: namely, grain of wheat was treated according to the patented method PCT n. PCT/IB2013/000797, further improved (Italian priority patent n. 102015000084813. Method for the detoxification of gluten proteins from grains of cereals and related medical uses filed on 17th December 2015. Inventor: Lamacchia C.). Specifically, 100 g of cleaned wheat grains was dampened until reaching 15–18% humidity, which was measured by a halogen thermal balance (Mettler Toledo, HB43-S, Switzerland), and subjected to rapid heating via microwaves (De’Longhi, Italy; approximately 1 min between 1000 and 750 watts), followed by slow evaporation of the water. The rapid heating and subsequent slow evaporation of the water was repeated until reaching a final temperature of 80–90°C, which was measured by a thermal camera (Fluke, i20 Model, Italy), and a moisture degree of 13–13.5% in the wheat grains.

After microwave treatment, the wheat kernels were cooled and dried at room temperature (24°C) for 12–24 h and then ground using an automatic laboratory mill MCKA (Bühler AG, Uzwil, Switzerland; diameter of grid 118–180 mm) (Bevilacqua et al., 2016b).

The flour produced by milling caryopses that had not been treated with microwaves was called control flour (CF). The particle size of the GFF and the CF used was in the range of 100 to 200 μm.

For all assays, fresh whole pasteurized homogenized cow’s milk (3.35 g/l protein; 5.00 g/l carbohydrates; 3.75 g/l fats) was used. Before each experiment, the viable count of milk was assessed to check that lactobacilli were below the detection limit (standard plate count).

#### Experimental Design

The optimization of the production of the synbiotic fermented milk was performed through a mixture design, called simplex centroid design: this kind of design involves three different variables; each variable is usually set at three different levels, identified with the code 0 (minimum), 1 (maximum), and 0.5 (half point of the range) (Bevilacqua et al., 2010a; Bevilacqua and Sinigaglia, 2010). In this research, the independent variables were the concentration of the flour (F), the inoculum of *L*. *acidophilus* (I), and the incubation temperature (T). More specifically, a design was developed using GFF (combinations A-F, design named GFA) and a second one using CF (combinations G-N, design named CFA).

**Table 1** reports the 12 combinations of the centroids and two controls (CNT-1 and CNT-2), i.e., two further combinations where no flour was added.

**Table 1:** Acidification of *L. acidophilus* La-5: fitting parameters of the lag-exponential equation (mean and SE).

Combination	Coded levels			Values			ΔpH_max_	d_max_	α	R
	I	F	T	I	F	T				
**GFA**										
A	1	0	0	8	0	30	2.73 ± 0.08	0.22 ± 0.01	–	0.978
B	0	1	0	4	5	30	1.68 ± 0.08	0.11 ± 0.01	2.00 ± 2.45	0.977
C	0	0	1	4	0	45	2.47 ± 0.11	0.14 ± 0.01	1.10 ± 2.63	0.978
D	0.5	0.5	0	6	2.5	30	2.59 ± 0.07	0.14 ± 0.01	1.89 ± 1.75	0.990
E	0.5	0	0.5	6	0	37.5	3.14 ± 0.03	0.24 ± 0.00	–	0.998
F	0	0.5	0.5	4	2.5	37.5	2.97 ± 0.10	0.21 ± 0.01	3.09 ± 2.04	0.982
**CFA**										
G	1	0	0	8	0	30	2.76 ± 0.07	0.20 ± 0.01	–	0.986
H	0	1	0	4	5	30	2.28 ± 0.09	0.12 ± 0.01	2.74 ± 2.31	0.984
I	0	0	1	4	0	45	2.58 ± 0.10	0.13 ± 0.01	1.21 ± 2.41	0.983
L	0.5	0.5	0	6	2.5	30	2.51 ± 0.08	0.14 ± 0.01	1.26 ± 2.03	0.987
M	0.5	0	0.5	6	0	37.5	2.98 ± 0.03	0.25 ± 0.00	–	0.998
N	0	0.5	0.5	4	2.5	37.5	2.94 ± 0.09	0.21 ± 0.01	2.68 ± 2.02	0.983
CNT-1	–	–	–	8	0	45	2.44 ± 0.08	0.21 ± 0.03	–	0.967
CNT-2	–	–	–	6	0	45	2.44 ± 0.07	0.17 ± 0.01	–	0.985

*I, inoculum (log cfu/ml); F, flour (g/l); and T, temperature, (°C). GFA, design with gluten friendly; CFA, design with control flour. ΔpH_max_, acidification (decrease of pH at the end of the assay); d_max_, acidification rate (ΔpH/day); α, time before the beginning of acidification (h); R, regression coefficient.*

#### Samples Preparation

According to the design, *L. acidophilus* was inoculated to 4–6–8 log cfu/ml in 15 ml of pasteurized milk supplemented with variable amounts of GFF or CF (0.0–2.5–5.0 g/l); then, the samples were incubated at 30, 37.5 or 45°C for 72 h. The analyses were done after 4, 6, 15, 18, 21, 24, 28, 30, 39, 48, and 72 h of incubation.

The acidification was monitored through pH measuring by a pH-meter (Crison, Barcelona, Spain).

#### Modeling

The experiments were performed in duplicate over two different samples; for each batch the measurements were repeated twice.

The data were modeled as acidification (ΔpH), i.e., pH decrease referred to the beginning of the experiment. ΔpH was used as the dependent variable for a primary modeling through the lag-exponential model by van Gerwen and Zwietering (1998) and by Baty and Delignette-Muller (2004), cast in the following form:

$$\Delta \text{pH}=\{\begin{array}{ll} 0 & t\leq \text{α}\\ \Delta {\text{pH}}_{\max}-\log\{1+(10^{\Delta {\text{pH}}_{\max}}-1) & t>\text{α}\\ \;*\exp[-{\text{d}}_{\max}(\text{t}-\text{α})]\} & \end{array}$$

where: ΔpH and t are the dependent and independent variables, respectively (acidification and time-h); α is the time before the beginning of the acidification kinetic (h); d_max_ is the maximal acidification rate (1/h); ΔpH_max_ is the maximum level of acidification.

When the acidification kinetic did not show the parameter α, the lag-exponential model was used as follows (Delignette-Muller et al., 2006):

$$\Delta \text{pH}=\Delta {\text{pH}}_{\max}-\log\{1+(10^{\Delta {\text{pH}}_{\max}}-1)*\exp(-d_{\max}t)\}$$

In a second step, ΔpH_max_ and d_max_ were used as input values for a multiple regression approach; the temperature, the level of inoculum and the amount of flour were used as independent variables. The analysis was done through the software Statistica for Windows (StatSoft, Tulsa, OK, United States), option Design of Experiments/mixture designs.

The model was built by using the option “quadratic,” for the evaluation of the individual (“Flour,” “Inoculum,” and “Temperature”) and interactive effects (“Flour ^∗^Inoculum,” “Flour ^∗^Temperature,” and “Temperature^∗^Inoculum”).

The most important output of the modeling was a polynomial equation reading as follows:

$$y=B_{0}+\sum B_{i}x_{i}+\sum B_{ij}x_{i}x_{j}$$

where, *y* and *x_i_* and *x_j_* are respectively, the dependent and the independent variables; *B_i_*, and *B_ij_* are the coefficients of the model. This model assessed the effects of linear (*x_i_*), and interactive terms (Σ*x_i_x_j_*) of the independent variables on the dependent variable.

The significance of the model was evaluated through the adjusted regression coefficients and the mean square residual, whereas the significance of each factor was assessed through the Fisher test (*P* < 0.05). A second output of the polynomial equation is the ternary plots.

The effect of each independent variable (inoculum, temperature, flour) on the fitting parameters of the acidification kinetic of *L. acidophilus* (ΔpH_max_ and d_max_) was evaluated through the individual desirability functions, estimated as follows:

$$\text{d}=\{\begin{array}{ll} 0, & \text{y}\leq {\text{y}}_{\min}\\ \left(\text{y}-{\text{y}}_{\min})/({\text{y}}_{\max}-{\text{y}}_{\min}\right) & {\text{y}}_{\min}\leq \text{y}\leq {\text{y}}_{\max}\\ 1, & \text{y}\geq {\text{y}}_{\max} \end{array}$$

Where y_min_ and y_max_ are the minimum and maximum values of the dependent variable, respectively.

The desirability was included in the range 0–1 (0 for the lowest value of ΔpH_max_ and d_max_ and 1 for their maximal values). The desirability profiles were built by setting the variables to the coded level 0.33 (inoculum to 5.3 log cfu/ml, temperature to 35°C, and flour to 1.65 g/l).

### Product Realization

#### Samples Preparation

Three different productions of functional fermented milk were realized as follows: one batch added with GFF (2.5 g/l) (GFA), another batch added with CF (2.5 g/l) (CFA) and a control batch without flour (LA). More specifically, fresh whole pasteurized homogenized cow’s milk was added with flours, inoculated with *L. acidophilus* at 6.5 log cfu/ml and left to ferment at 37°C for 2 days. The fermentation was monitored by measuring the pH through a pH electrode 50^∗^50T CRISON (Crison Instruments, Barcelona, Spain). After the fermentation, the samples were stored at 4°C for 90 days; microbiological and sensorial analyses, measurements of pH, a_*w*_ and color were made as detailed below.

For microbiological analyses the following media were used: MRS Agar (MRSA) acidified to pH 5, incubated at 37°C under anaerobiosis for *L. acidophilus*; MRSA incubated at 30 and 42°C for 48 h under anaerobiosis, for mesophilic and thermophilic lactobacilli, respectively; M17 incubated at 30 and 42°C for 48 h under anaerobiosis, for lactococci and streptococci, respectively; Slanetz/Bartley Agar incubated at 37°C for 48 h, for enterococci; Plate Count Agar (PCA) incubated at 5°C for a week or 32°C for 48 h for psychrotrophic bacteria and mesophilic bacteria, respectively; Baird-Parker agar base, with egg yolk tellurite emulsion, incubated at 37°C for 48 h for staphylococci and *Micrococcaceae*; Pseudomonas Agar Base (PAB) with CFC Selective Supplement incubated at 25°C for 48 h for *Pseudomonas* spp.; Violet Red Bile Glucose Agar (VRBGA), incubated at 37°C for 24 h for *Enterobacteriaceae;* Violet Red Bile Agar (VRBA) incubated at 37°C or 42°C for 18–24 h for total and fecal coliforms, respectively; Sabouraud dextrose agar, supplemented with chloramphenicol (0.1 g/l) (C. Erba, Milan, Italy), incubated at 25°C for 48 h or 5 days, for yeasts and molds, respectively. All the media and the supplements were from Oxoid.

The viable count of *L. acidophilus* was confirmed by a random isolation of some colonies, microscopic, phenotypic tests and PCR analysis (Speranza et al., 2015).

At each sampling time, pH values were measured in duplicate by a pH-meter and a_*w*_ values were measured in triplicate by an AQUALAB CX-2 (Decagon Device, Pullman, WA, United States). Color was evaluated by a colorimeter Chroma Meter (Minolta, Japan) by measuring CIE L^∗^ (lightness), a^∗^ (redness) and b^∗^ (yellowness) values.

The sensory evaluation panel consisted of 15 panelists aging between 22 and 38 years (students and researchers of the Department of the Science of Agriculture, Food and Environment (SAFE), University of Foggia). Using a scale ranging from 0 to 10 (where 0 stands for the most attractive attributes and 0 for the absolutely unpleasant attributes), the sensorial overall quality of the samples was determined by evaluating color, odor and overall acceptability.

#### Modeling

The experiments were repeated twice on two independent samples. The results of *L. acidophilus* were modeled as decrease of viable count over the time and fitted through the lag-exponential model, as reported above for the acidification kinetic.

The sensory scores were analyzed through the non-parametric test of Kruskal-Wallis (Analysis of Variance by ranks); the critical value of P was set to 0.05.

## Results

### First Phase: Product Optimization

The first step was a product optimization to design a synbiotic active drink, fermented with *L. acidophilus* La-5 and containing GFF as a beneficial ingredient. At this scope, the screening was aimed at choosing the optimal conditions to produce the active drink, in terms of level of inoculum, temperature and amount of flour. Therefore, two different designs were performed: the first with the control flour (design CFA) and the second one with the GFF (design GFA).

A requisite to design a synbiotic food is that the prebiotic component/beneficial ingredient must not affect the performances of the starter and/or probiotic microorganisms; thus, the acidification of *L. acidophilus* was assessed in presence of flour. **Table 1** shows the combinations of the design and the performances of the probiotic as ΔpH_max_ (maximum acidification) and d_max_ (acidification rate). In the design with GFF, ΔpH varied from 1.68 to 3.14 and the acidification rate from 0.11 to 0.24 h^−1^. Similar results were found for the CF (acidification in the range 2.28–2.98 and rate 0.12–0.25 h^−1^).

**FIGURE 1:**
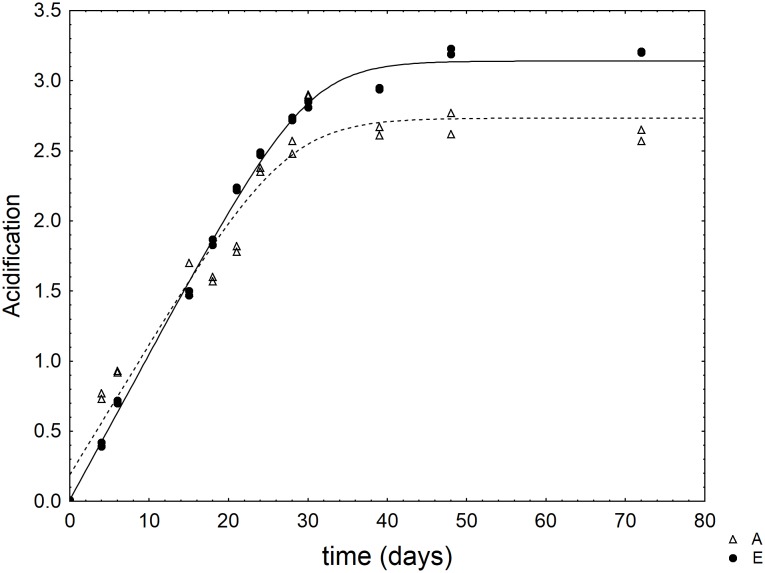
Kinetic of acidification of *L. acidophilus* in some selected combinations of the mixture design (**Table 1**). The lines represent the best fit through the lag-exponential model.

As an example, **Figure 1** shows the kinetic of acidification in two selected combinations of the design.

In the second step of the screening, ΔpH_max_ and d_max_ were used as dependent variables for a multiple regression approach. The first output was the table of the standardized effects, showing the statistical weight and the significance of each individual (flour, inoculum, and temperature) and interactive factors. All variables were significant as individual terms on both ΔpH_max_ and d_max_; however, the most significant factor was the level of inoculum, followed by the temperature and finally by the amount of flour.

ΔpH_max_ and d_max_ were also affected by the interactive terms and generally the strongest weight was found for the interaction “flour^∗^temperature” (**Table 2**).

**Table 2:** Standardized effects for flour, inoculum, and temperature on the maximum acidification (ΔpH_max_) and acidification rate (d_max_) of *L. acidophilus* in presence of Gluten Friendly (design GFA) and control flour (design CFA).

	ΔpH_max_		d_max_	
	GFA	FCA	GFA	FCA
Inoculum	57.878	58.610	33.734	35.784
Flour	35.500	48.367	17.393	20.985
Temperature	52.297	54.864	22.194	23.562
Inoculum^∗^flour	6.676	−^∗^	−2.478	−2.864
Inoculum^∗^temperature	9.367	5.472	7.922	11.454
Flour^∗^temperature	15.484	8.805	10.499	11.392
R_ad_^2^	0.972	0.903	0.949	0.952

*R_ad_^2^, determination coefficient adjusted for the multiple regression. ^∗^Not significant.*

**FIGURE 2:**
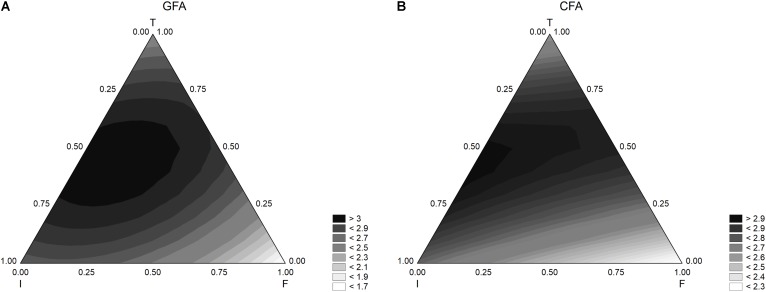
Triangular surfaces for the effect of flour (F), inoculum (I), and temperature (T) on the acidification (parameter ΔpH_max_) performed by *L. acidophilus*. **(A)** GFA, design with gluten friendly flour; **(B)** CFA, design with control flour.

Other outputs of a mixture design are the ternary plots, showing the interactions of three factors in a bi-dimensional space. For the sample GFA (containing GFF), the model predicted the maximum extent of acidification at the coded level 0.5 of inoculum and temperature (6 log cfu/ml and 37°C) and with an amount of flour in the range 0.0–0.50 (from 0 to 2.50 g/l) (**Figure 2A**). Similar results were found for the acidification in presence of the control flour (CFA) (**Figure 2B**), although the effect of flour seemed stronger and the acidification was lower at the coded level 0.25 (1.25 g/l).

**FIGURE 3:**
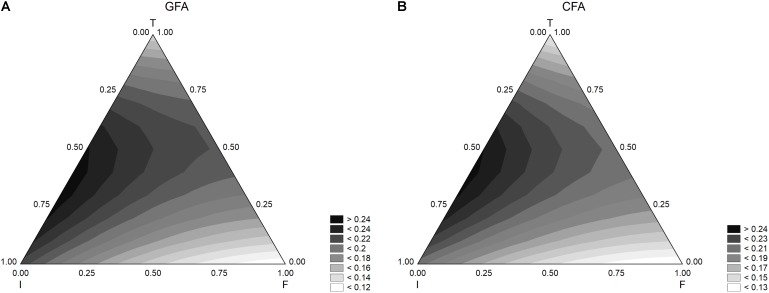
Triangular surfaces for the effect of flour (F), inoculum (I), and temperature (T) on the acidification rate (parameter d_max_) by *L. acidophilus*. **(A)** GFA, design with gluten friendly flour; **(B)** CFA, design with control flour.

**Figure 3** shows the results for the acidification rate. The model predicted the highest values of this parameter when both inoculum and temperature were at the coded levels 0.5.

A ternary plot is an important tool; however, it could not be used to analyze the quantitative effect of each individual term. A solution to counteract this limit is the use of the desirability approach.

The desirability is a dimensionless parameter, ranging from 0 to 1 and is the answer to question: how much desired is an output? The reply is: 0 for the worst result (the lowest values of acidification and acidification rate) and 1 for the best one (the highest values of acidification and acidification rate). Moreover, a desirability profile is often completed by a prediction profile, which shows the predicted values of the dependent variable as a function of the coded values of the factors of the design.

**FIGURE 4:**
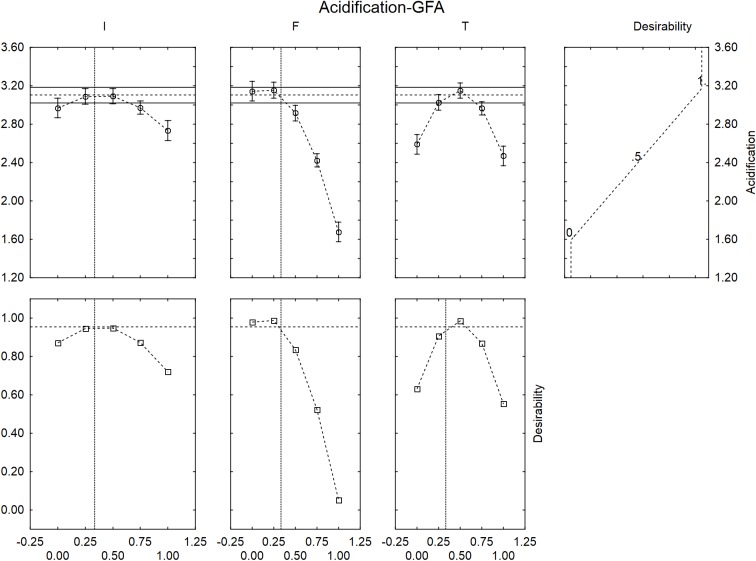
Prediction (upper side) and desirability profiles (down side) for the effect of inoculum (I), Gluten Friendly flour (F), and temperature (T) on the acidification (ΔpH_max_) performed by *L. acidophilus*.

**Figure 4** shows the desirability profiles for ΔpH_max_ in presence of GFF; the effect of the level of inoculum was not strictly linear but quadratic. An increase of the level of inoculum, in fact, could exert a negative effect of the extent of acidification with a decreased performance of *L. acidophilus* at the coded level 1 (inoculum at 8 log cfu/ml). A quadratic effect was also found for the temperature, with a decreased effect at the coded levels 0 and 1 (30 and 45°C).

The correlation “flour vs. acidification” was negative; the model predicted the highest value of acidification for the coded levels 0 and 0.25 (0 and 1.25 g/l) and a level of acidification nearby the optimal value (ca. 3) at the coded level 0.5 (2.5 g/l of GFF); on the other hand, the extent of acidification strongly decreased at the coded levels 0.75 and 1.0, thus suggesting a negative effect of the flour on the performances of the probiotic.

**FIGURE 5:**
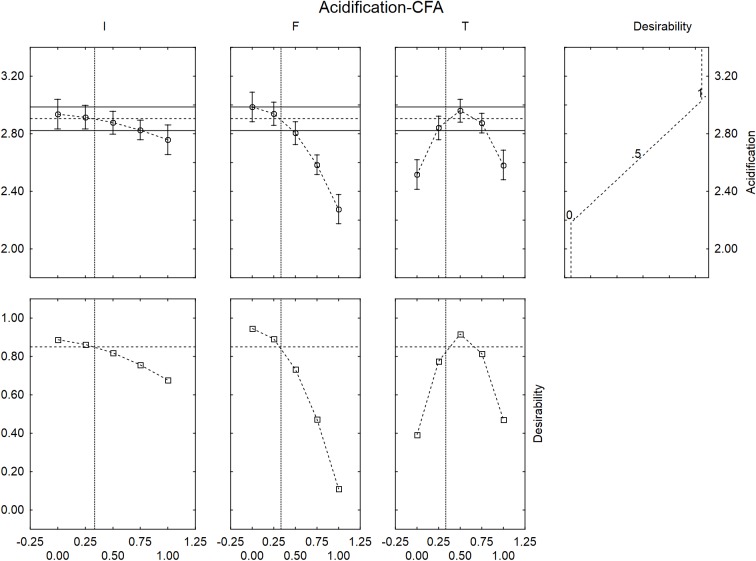
Prediction (upper side) and desirability profiles (down side) for the effect of inoculum (I), control flour (F), and temperature (T) on the acidification (ΔpH_max_) performed by *L. acidophilus*.

The desirability profiles showed similar trends in presence of the control flour (**Figure 5**), with a negative effect on the acidification for an inoculum of 8.0 log cfu/ml, an amount of flour >2.5 g/l and a temperature at 30 and 45°C.

The desirability profiles of d_max_ for both GFF and CF suggested the same results (data not shown).

The outputs of the desirability and prediction profiles were used to design/optimize the conditions to produce the active drink; thus, the variables for the fermentation of milk were set as follows:

• *L. acidophilus* at 6.5 log cfu/ml
• Flour at 2.5 g/l
• Temperature at 37°C.

### Second Phase: Product Realization

After the fermentation the viable count of probiotic was 8.30 log cfu/ml, the pH 4.11 (LA) and 4.0 (CFA and GFA) and the A_*w*_ 0.990.

**FIGURE 6:**
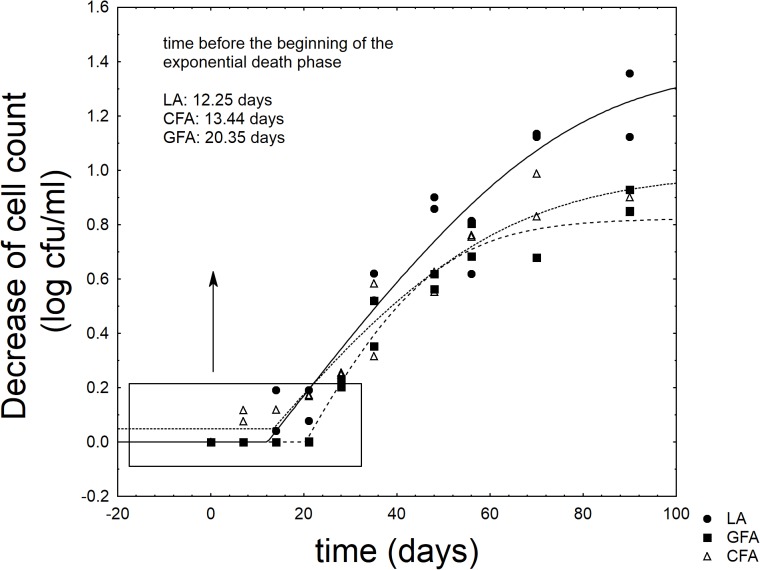
Decrease of cell count of *L. acidophilus* in the fermented milk. LA, product with *L. acidophilus*; GFA, product with *L. acidophilus* and Gluten Friendly flour; and CFA, product with *L. acidophilus* and control flour. The line represents the best fit through the lag-exponential model.

**Figure 6** shows the results for the viable count of *L. acidophilus* throughout the refrigerated storage of the synbiotic drink. The probiotic never attained the critical level (7 log cfu/ml) (Rosburg et al., 2010) (data not shown); however, the supplementation of GFF exerted a significant effect on the shape of the death kinetic. The probiotic experienced a shoulder length (SL), i.e., a time before the beginning of the exponential death kinetic; this parameter was significantly affected by the formulation. The probiotic alone (sample LA) experienced a SL of 11.25 days and the addition of control flour did not act on it (FCA, 13.44 days) (*P* > 0.05). On the other hand, the supplementation of GFF significantly prolonged SL and increased it to 20.35 days (sample GFA) (*P* < 0.05).

Concerning the other microbiological results, the levels of enterobacteria, pseudomonads, yeasts and molds, enterococci and psychrotrophic bacteria were always below the detection limit (data not shown). Both pH and Aw did not undergo significant changes throughout storage (data not shown).

**FIGURE 7:**
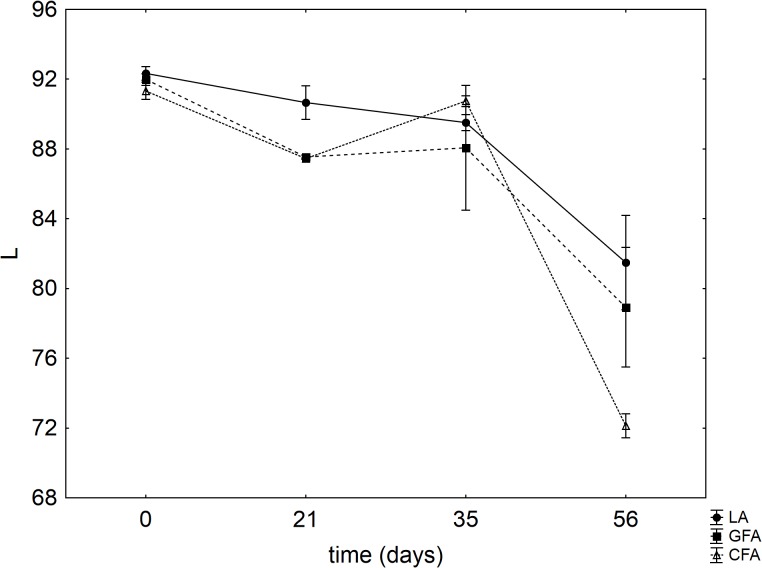
Evolution of L (mean values ± SE). LA, product with *L. acidophilus*; GFA, product with *L. acidophilus* and Gluten Friendly flour; and CFA, product with *L. acidophilus* and control flour. The numbers after the acronym of the samples indicate the days of storage (analysis after 7, 14, 21, 28, 35, and 56 days).

**Figure 7** shows the results for the instrumental color evaluation (luminosity, L). A significant decrease of L was found after 56 days and this trend was more pronounced in the sample CFA, containing the control flour.

**FIGURE 8:**
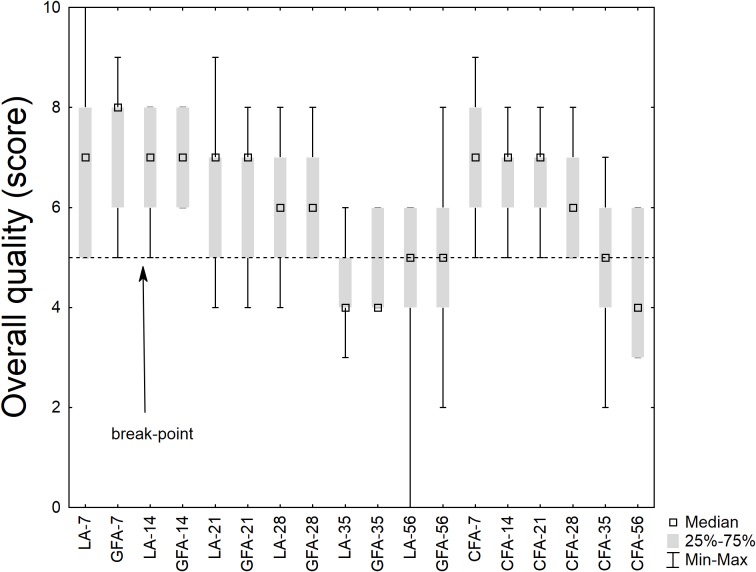
Box-whisker plot on the sensory score (overall quality). LA, product with *L. acidophilus*; GFA, product with *L. acidophilus* and Gluten Friendly flour; and CFA, product with *L. acidophilus* and control flour. The numbers after the acronym of the samples indicate the days of storage (analysis after 7, 14, 21, 28, 35, and 56 days).

Concerning the sensory scores, **Figure 8** shows the results for the overall quality; the results were analyzed through a non-parametric test, as they did not fit with the basic requisite of a parametric statistic (normal distribution). The median score was higher than the break-point (score, 5) for 28 days; then it decreased. However, the differences amongst the samples were not significant.

## Discussion

The starting point of this research was a patent about the use of a novel temperature-based method on wheat kernels to induce structural changes in gluten proteins (Gluten Friendly^TM^) (Lamacchia et al., 2013, 2015). Some investigations performed on this approach also focused on the effect of the bread produced with GFF on some probiotic and foodborne strains (*L. acidophilus*, *Bifidobacterium animalis* subsp. *lactis*, *Staphylococcus aureus*, and *Salmonella* Typhimurium) to pinpoint a potential modification of the survival of these selected targets under strict controlled conditions: in particular, the study was conducted to determine whether GFF could have a beneficial effect by modifying the qualitative-quantitative composition of gut microbiota (Bevilacqua et al., 2016b). The results of the mentioned study highlighted a protective effect on *L. acidophilus* viability observing a significant lowering of its death rate and a prolongation of the survival time from 70.28 to 93.46 h with 0.8 g/l of GFF bread; therefore, the idea to design a synbiotic fermented milk containing GFF as a beneficial ingredient and *L. acidophilus* as functional starter was exploited in this experimentation. During the design of a synbiotic food, it is mainly important that the prebiotic component must not affect the performances of the starter and/or probiotic microorganisms; thus, the first phase of this research was performed to assess the acidification of *L. acidophilus* in presence of flour and as a function of the level of inoculum and temperature to optimize the conditions to produce the active drink. Since a negative effect on the performances of the strain was observed for an inoculum of 8.0 log cfu/ml, an amount of flour >2.5 g/l and a temperature at 30 and 45°C, the fermented milk was produced by inoculating *L. acidophilus* at 6.5 log cfu/ml, adding flour at 2.5 g/l and performing the fermentation at 37°C. During the refrigerated storage of the synbiotic drink produced, the obtained results confirmed those obtained by Bevilacqua et al. (2016b); in particular, the positive effect of GFF on the death kinetic of the probiotic was evidenced by a significant prolongation of the shoulder length of the test microorganism (to 20.35 days), thus confirming the lowering of the death rate as previously reported. As reported in literature, prebiotics could act toward lactobacilli through three possible ways: effect on metabolism, induction of a higher stability of membrane and starvation. For example, fructooligosaccharides (FOSs) are reported to affect the regulation of genes involved in the primary metabolism (fructokinase, phosphoenolpyruvate transport system, β-fructofuranosidase, and α-glucosidase) and/or on the regulation of genes linked to the synthesis of fatty acids, proteins and cell wall (Saulnier et al., 2007). On the other hand, some studies reported a protective effect of fructans (inulin and FOS) by inducing a higher stability of membrane against some stresses (freezing, dehydration, etc.) (Vereyken et al., 2001, 2003a,b), due to a probable interaction with the phospholipids of the membrane (Schwab et al., 2007). The third way of action was observed by Altieri et al. (2011) which suggested the induction of a kind of starvation when *L. plantarum* was grown in a medium containing inulin or FOS; this effect was also suggested by Saulnier et al. (2007), Hussain et al. (2009), Bevilacqua et al. (2010b, 2016a,c) and by Wang et al. (2011) which observed an increase in viability as practical output of starvation.

The data collected on GFF suggest that it cannot be simply labeled as a prebiotic (Bevilacqua et al., 2016b; Lamacchia et al., 2018). The protective effect observed for GFF could be considered quite different from those exerted by some prebiotics, because it probably did not induce resistance in cells but lowered the death rate. This effect was previously observed in some cell-free filtrates or bifidogenic factors which enhanced growth by altering membrane permeability, combating cell aging, etc. (Oda et al., 2013; Kang et al., 2015). These bifidogenic factors are different from the traditional prebiotics because of their nature (peptides or proteins).

A way to elucidate and explain the effect of GFF is in its technology. As supposed elsewhere (Bevilacqua et al., 2016b; Lamacchia et al., 2016), the alternation of high temperatures to evaporation phases induces different spatial conformation of the amino acid sequences in hydrated wheat caryopses treated with the Gluten Friendly^TM^ technology, inducing a rearrangement of the secondary and tertiary structure of the gluten proteins.

This rearrangement was suggested as the main cause for an exposure of the positive charge, which in turn could be responsible of an interaction with the teichoic acids of the cell wall of lactobacilli. This interaction could lead to a protection of the cell from aging, and/or a change in the membrane permeability (Bevilacqua et al., 2016b). This effect was later found and recovered on the lactobacilli of the fecal microbiota (Costabile et al., 2017).

The idea of positive charges was indirectly confirmed by the different bioactivity toward lactobacilli and *Salmonella* sp., a Gram negative (Bevilacqua et al., 2016b). *Salmonella* possesses the outer membrane, and different distribution of charges. Moreover, the distribution of charges, probably associated with teichoic acids, could explain the quick effect on lactobacilli and the delayed effect on bifidobacterial.

Another possible idea, suggested by some preliminary evidences, is a possible use of the modified gluten as an alternative source of nutrients by lactobacilli under stressful conditions; however, this hypothesis should be confirmed by *ad hoc* investigations. Maybe, the mode of action is in the middle with a combination of a positive effect on permeability and the use as nutrient.

An important requisite in the design of a synbiotic food is that the probiotic concentration at the time of sale was higher than a break-point fixed in 10^6^ cfu/g or cfu/ml by the Italian legislation (Fortina, 2007) and recently increased to 10^7^ cfu/g or 10^9^ per day (Rosburg et al., 2010; Italian Ministry of Health, 2018). Our results showed that the population of *L. acidophilus* throughout the refrigerated storage of the synbiotic drink never attained the critical level (7 log cfu/ml) and no adverse effects on organoleptic characteristics of the product were exerted by both the probiotic organism and the prebiotic addition.

The novelty of this paper is a structured statistical approach on the quantitative effects of GFF on the survival of *L. acidophilus* La-5 and represents the first step to set up and design a synbiotic fermented milk, combining this probiotic and GFF. The practical implication of the results could be summarized as follows:

(1) The flour could be used at a maximum concentration of 2.5 g/l otherwise it could negatively affect the acidification kinetic.
(2) The supplementation of GFF exerted a positive effect on the viability of the probiotic, with a prolongation of the shoulder length to 20 days, whereas in the control or in presence of CF the shoulder length was ca. 12–13 days.
(3) The use of GFF did not act on the sensory scores and on the physico-chemical parameters.
(4) The idea of a functional product combining a probiotic and GFF is technologically feasible. The benefits and the added value of this new product is the possibility of using an ingredient able to exert a dual effect: (i) to act in the product by increasing the viability of the probiotic (economic value and prolonged shelf life) (results of this paper); (ii) to positively modulate the microbiota of celiac and healthy people (functional effect) (results of the previous researches).

Some evidences suggested that the increased survival of *L. acidophilus* could be the result of a shift of the death curve with a prolonged shoulder length; however, further experiments are required to try to elucidate the molecular mechanisms beyond it (change in the permeability of the membrane, possible use of the modified gluten as an alternative nutrient source or other mechanisms).

In conclusion, this research aimed at designing an approach and a method to combine GF with probiotics in fermented milk and *L. acidophilus* La-5 was used as the test microorganism; the results of this research showed the suitability of this method and suggested the possibility of using it for many other strains.

## Author Contributions

MC, MS, and CL conceived the study. AB, BS, DC, and MC designed the experiments. CL and DM prepared the GFF. BS, DC, and DM performed the experiments. AB performed the statistic. BS and AB wrote the manuscript. All authors interpreted the results and reviewed the paper. CL funded the research.

## Disclaimer

This publication reflects only the author’s view and the Agency is not responsible for any use that may be made of the information it contains.

## Conflict of Interest Statement

CL declares to be the inventor of the following patents “method for the detoxification of gluten proteins from grains of cereals. Patent Cooperation Treaty PCT/IB2013/000797” and “methods for the detoxification of gluten proteins from grains of cereals and related medical uses. Italian priority patent n° 102015000084813 filed on 17.12.15.” The remaining authors declare that the research was conducted in the absence of any commercial or financial relationships that could be construed as a potential conflict of interest. Casillo Group had no role in the design of the basic patent of this research (Gluten Friendly^TM^ temperature-based process), and did not play any role in the design of this research.
